# Impaired functional capacity of polarised neonatal macrophages

**DOI:** 10.1038/s41598-019-56928-4

**Published:** 2020-01-17

**Authors:** Stephan Dreschers, Kim Ohl, Nora Schulte, Klaus Tenbrock, Thorsten W. Orlikowsky

**Affiliations:** 1 Section of Neonatology, University Children’s Hospital, Aachen, 52074 Germany; 2 Department of Pediatrics, RWTH Aachen University, Aachen, Germany

**Keywords:** Infection, Pattern recognition receptors

## Abstract

Neonatal sepsis is accompanied by impaired apoptotic depletion of monocytes and macrophages (MΦ), aberrant cytokine production, impaired cell metabolism, and sustained inflammation. Macrophage-colony stimulating factor (M-CSF) triggers the differentiation from monocytes into MΦ (MΦ-0). Interleukin-10 (IL10) and Interferon-gamma (IFNy) further differentiate MΦ subpopulations, the anti-inflammatory MΦ-IL10 and the pro-inflammatory MΦ-IFNy subtype. We previously have shown significant differences between adult (PBMΦ) and cord blood (CBMΦ) in the metabolism of all subtypes. To test the hypothesis whether the competence to differentiate monocytes into MΦ-0 and to polarise into MΦ-IFNy and MΦ-IL10 was diminished in CBMΦ as compared to PBMΦ, we polarised monocytes by cultivation with M-CSF for 72 h, followed by stimulation with IFNy or IL10, for 48 h. After flow cytometry based immunotyping, we tested four functions: Phagocytosis of GFP-*E. coli*, uptake of erythrocytes, T-cell proliferation, induction of regulatory T-cells as well as phosphorylation analysis of AKT and STAT1/STAT3. Phosphorylation of STAT-1 and STAT-3, obligatory to differentiate into MΦ-IFNγ, MΦ-0 and MΦ-IL10, was found to be aberrant in CBMΦ. Whereas infected MΦ-0 showed identical phagocytic indices and intracellular degradation, TLR4-expression, NFkB up-regulation, IL10-, IL6-, and TNFα production of CBMΦ-0 were reduced. In addition, the capacity to bind aged erythrocytes and the consecutive IL10 production was lower in CBMΦ-IL10. Polarised PBMΦ-IFNy and PBMΦ-IL10 expressed higher levels of co-stimulatory receptors (CD80, CD86), had a higher capacity to stimulate T-cells and induced higher amounts of regulatory T-cells (all p < 0.05 vs. corresponding CBMΦ). Hypoxia-inducible-factor-1α (HIF-1α) was stronger expressed in CBMΦ-IFNy and upregulated in infected CBMΦ-0, whereas heme-oxygenase 1 (HO-1) expression was similar to adult PBMΦ. Neonatal MΦ-0, MΦ-IFNy and MΦ-IL10 polarisation is impaired with respect to phenotype and functions tested which may contribute to sustained inflammation in neonatal sepsis.

## Introduction

Macrophages (MФ) have an extremely broad spectrum of functions in both the induction and resolution of inflammation due to their heterogeneity and plasticity. They are involved in a range of processes, including tissue homeostasis, clearance or pulmonary surfactant as well as inflammation. Deriving from monocytes, they develop into different populations of circulating inflammatory or resident macrophages (MФ) recruited to various tissues. These processes occur in response to inflammation or infection but also in homeostasis in order to replace apoptotic (MФ)^1^. The migration of monocytes to the site of infection is crucial^2^. After infiltrating the infected tissue they polarise into functional distinguishable MФ. The term “polarisation” instead of differentiation became accepted, since this process depends on the local cytokine milieu and specific microenvironmental cues, and therefore is reversible^3,4^.

By analogy to T helper cell (Th) nomenclature, MФ have been classified into two categories: type 1 or classically activated (M1-) and type 2 or alternatively activated M2-MΦ. *In-vitro*, peripheral monocytes can be polarised by M-CSF, GM-CSF or G-CSF into non-polarised MФ^5^. M1 type MФ originate from monocytes stimulated with GM-CSF or M-CSF in the presence of IFN-y and/or bacterial products. In contrast, M2 type MФ are polarised by M-CSF and IL-4 or IL-13 (M2a), immune complexes together with lipopolysaccharide (LPS) or IL-1ß (M2b), and M CSF and IL10 or glucocorticoids (GC) respectively (M2c), reviewed in^6^.

M1 and M2 type MΦ can be roughly categorized regarding their function. M1-MΦ display high microbicidal activity and suppress tumor growth, whereas M2-MΦ are characterized by an enhanced phagocytic capacity which is shaped to eliminate cellular and apoptotic debris rather than to neutralize pathogens. This, so called, M1/M2 paradigm was refaced, since it became difficult to classify MΦ subsets comprehensively^7^.

From the transcriptional level, administration of LPS or IFNy dramatically activates up to 90% of genes giving raise to M1-MΦ. These M1-MΦ are polarised from monocytes, which are - under physiological conditions – constantly exposed to M-CSF establishing naïve, “standby” MΦ. In this manuscript they are termed MΦ-0.

In M2-MΦ subsets transcriptome changes are more distinct. Therefore, todays‘ nomenclature is based on more distinct “sensor” and “effector” molecule expressions^8^. According to the expression of receptors, intracellular factors and secreted cytokines, M2-MΦ subsets are renamed to MΦ-IL4/MΦ-IL13 (replacing M2a), MΦ-IC (replacing M2b) and MΦ-IL10 (replacing M2c). This nomenclature is used in the present manuscript.

Upon infection, MΦ-0 are polarised by pathogen-activation-pattern-molecules (PAMPs) to MΦ-IFNy, exhibiting a “kill/fight” status, which is accompanied with secretion of inflammatory cytokines, reactive-oxygen-status (ROS) and nitric oxide (NO). After neutralization of the pathogens a substantial reprogramming of MΦ-IFNy starts to skew pro- to anti-inflammatory responses. The MΦ status is set to “fix/heal”^9^. The M2-MΦ are also reported to be important for matrix deposition and tissue remodeling^5^.

This functional transition of MΦ subpopulations is a crucial phase to avoid the individual of being harmed by either incomplete neutralization of pathogens or sustained inflammation. Compared to adults the immune system of newborns is less experienced to execute this transition to resolve inflammation and therefore more vulnerable^10^.

Reports concerning MΦ polarisation from neonatal monocytes in *ex-vivo* setups are rare. We have previously shown that CBMФ exhibit reduced expression of phagocytosis receptors and cytokines in addition to altered energy metabolism. In particular, IFNy as well as IL10 activated CBMФ completely fail to increase glycolysis and furthermore show reduced activation of the mTOR pathway, which is important for survival in sepsis^11^. Reduced polarisation capacity is likely to suppress MФ functions in neonates, such as activation and expansion of specialized T-cell subpopulations. In line with this observation, they were found to be less efficient in antigen presentation^12^. We thereby observed that scavenger receptors, e.g. CD163, and Fc receptors, critically involved in phagocytosis of bacteria and cellular debris, i.e. elimination of haemoglobin-haptoglobin complexes (Hb:Hp), are overexpressed in MΦ-IL10 from adults (PBMΦ-IL10) but not in newborns (CBMΦ-IL10)^1,11^.

An aberrant polarisation of CBMΦ can also be caused by immune cell populations specific for the neonatal period of life. CD71^+^ erythroid cells as well as myeloid derived suppressor cells (MDSCs) were described to reduce pro-inflammatory processes after bacterial infections^13,14^. The exact role of CD71^+^ erythroid cells and MDSCs is still controversial, since the newborn can either benefit or be harmed from effects maintained by these cells.

MΦ polarisation attracted interest, because the development of therapeutical strategies could benefit from a temporal programming of immune cells. This is especially true for MΦ-IL10, which can be polarised by administration of GC. Recent publications reported an increase in M2-MΦ after GC treatment and an improved outcome in acute lung injury^15^. Patients with therapeutically polarised MΦ-IL10 recovered with a better outcome from asthma^16^. MΦ are a target in neonatal hypoxic ischemic encephalopathy (HIE) for being programmed to M2- MΦ^17^.

Here we tested the hypothesis that CBMФ exhibit reduced phenotypic and functional characteristics in comparison to PBMФ. We have previously shown that CBMФ are less responsive to polarise further into CBMФ-IL10, thus exhibiting a higher risk to contribute to sustained inflammation.

To this end, we compared the expression of surface markers on MΦ-0 and MΦ-IL10, derived from either cord blood or peripheral blood of adult donors. Furthermore, we investigated the expression of the intracellular signal transducers STAT1/STAT3 and PI3K/AKT, which are engaged in infection-induced signaling via TLR4 and contribute to cytokine- as well as CD163 expression. We quantified HIF-1α and HO-1 levels, which link primary immune responses like pathogen-associated-molecular pattern (PAMP) recognition, cytokine production and metabolism. Finally, we analysed the MΦ-dependent T cell activation and induction of regulatory T cells.

## Results

### Activated STAT-kinase expression drives polarisation of PBMΦ but is impaired in CBMΦ subsets

We cultivated monocytes (either peripheral blood monocytes (PBMO) or cord blood monocytes (CBMO) under conditions, which differentiate these cells into MΦ-0 and further lead to their development into either pro-inflammatory MΦ-IFNy or anti-inflammatory MΦ subsets such as MΦ-IL4, MΦ-IL10 and MΦ-IL13.

**Figure 1:**
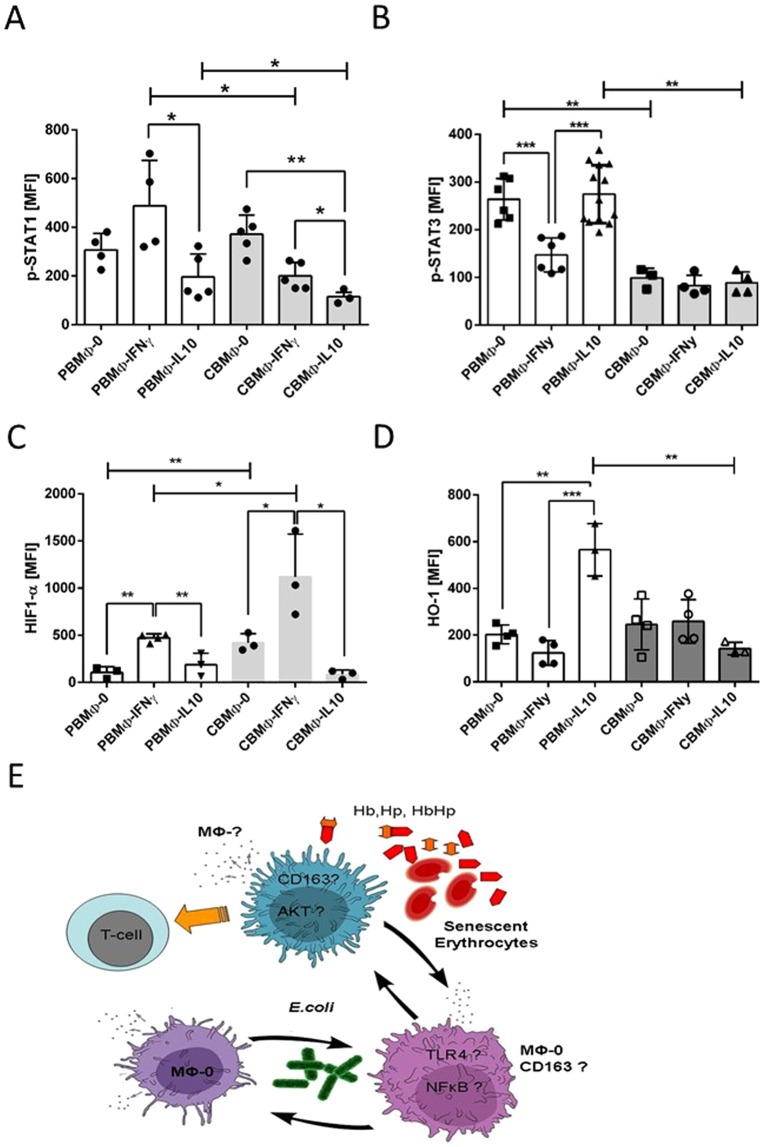
STAT-1/STAT-3 phosphorylation status in MΦ subsets. MΦ subsets were further analysed for STAT-1 (**A**) and STAT-3 phosphorylation (**B**), Furthermore, the intracellular levels of HIF-1α (**C**) and HO-1 were determined (**D**, *p < 0.05, **p < 0.01, ***p < 0.005; bars, student’s t-test, blunt ended bars, two-way-ANOVA). Sketch (**E**) summarizes the investigations regarding to MΦ and T cells and the indicated stimuli. Cytokine release of MΦ is represented by dots and small arrows. Question marks represent cellular factors whose expression will be examined in Figs. 2–5.

We extended our previous studies by assessing the phosphorylation status of the intracellular signal transducers STAT-1 and STAT-3, which have been shown to be obligatory for proper polarisation to the subtypes of MΦ-IFNy, MΦ-0 and MΦ-IL10^7^ (Fig. 1A,B). Under IFNy cultivation, PBMΦ showed highest STAT1 phosphorylation and lower STAT-1 phosphorylation in MΦ-0 and MΦ-IL10, as already described^7^. In contrast, CBMΦ exhibited an aberrant STAT-1 phosphorylation profile: Whereas the MΦ-0 type showed a comparable STAT-1 phosphorylation to adult PBMΦ, CBMΦ-IFNy and CBMΦ-IL10 displayed less STAT-1 phosphorylation (Fig. 1A).

STAT3 phosphorylation was significantly stronger in PBMΦ-0 and in PBMΦ-IL10 compared to corresponding CBMΦ subtypes (Fig. 1B). Taken together, PBMΦ responded to cytokine driven polarisation with higher levels of activated STAT1 in PBMΦ-IFNy and higher activated STAT3 in PBMΦ-IL10.

Next, we analysed two nuclear factors, hypoxia-inducible factor-1alpha (HIF-1α) and heme-oxygenase-1 (HO-1). HIF-1α was most highly expressed in PBMΦ-IFNy (Fig. 1C). PBMΦ-0 and PBMΦ-IL10 contained lower intracellular HIF-1α concentrations. CBMΦ exhibited a comparable expression profile, however the expression of HIF-1α in both, CBMΦ-0 and CBMΦ-IFNy was two times higher compared to their adult counterparts. The anti-inflammatory HO-1 was found expressed most strongly in PBMΦ-IL10 and CBMΦ-0 (Fig. 1D). In summary, the results suggest that MΦ from aldults polarised as previously shown and expected, whereas CBMΦ exhibited an aberrant polarisation profile.

In order to analyse functional consequences, we challenged CBMΦ-0 and CBMΦ-IL10 with *Escherichia coli* (*E. coli)*, or Hb:Hp complexes from senescent erythrocytes (EL). In particular, we focused on keyplayers of TLR4/NFĸB and CD163/AKT/IL10 signaling pathway (see sketch Fig. 1E).

### Phagocytosis, intracellular killing, and cytokine production of infected MΦ-0

**Figure 2:**
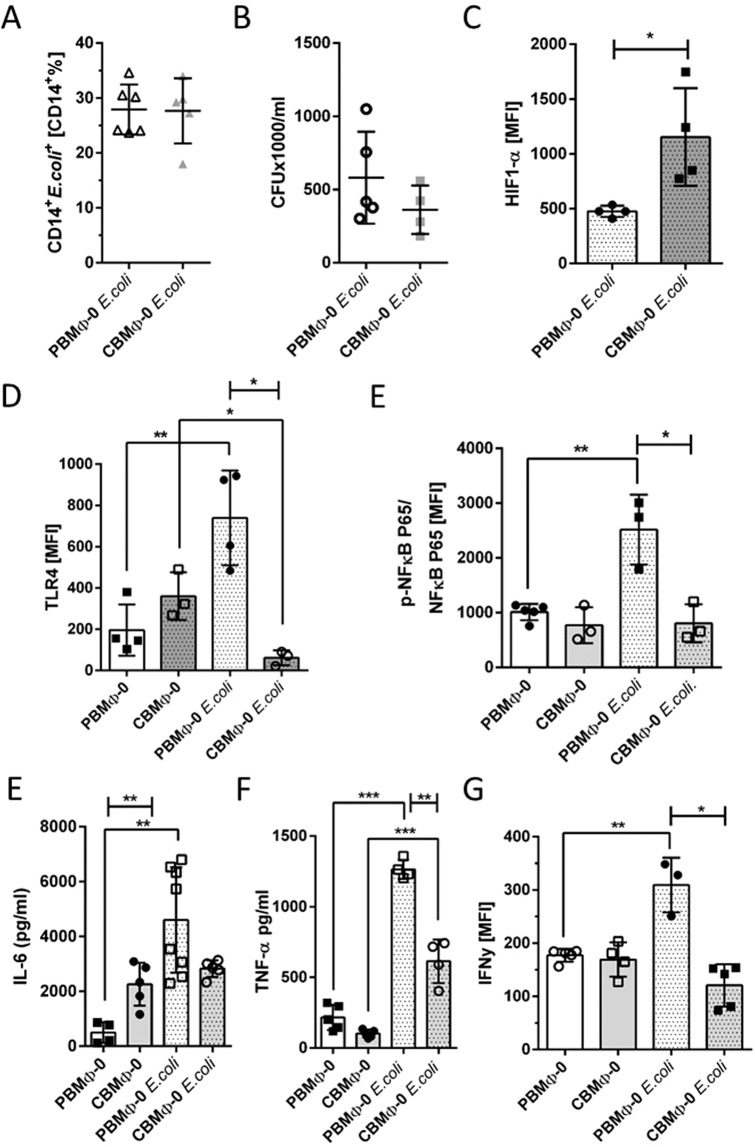
Periphagocytic reactions after infection with *E. coli*. Phagocytic indices of indicated MΦ were assessed 4 h p.i. (post infection; **A**). Killing of *E. coli* was assessed 12 h p.i. (**B**). HIF-1α expression was measured 12 h p.i. (**C**). Expression of TLR-4 (**D**) and phosphorylated NFκB-P65 (**E**) were assessed by FACS 4 h p.i. Secretion of IL6 (**F**) and TNFα (**G**) were determined by ELISA 12 h p.i. Intracellular production of IFNy (**H**) was also assessed (**G**, all charts, *p < 0.05, **p < 0.01, ***p < 0.005; bars, student’s t-test, blunt-ended bars, two-way-ANOVA).

In order to simulate the impact of sepsis, we infected (non-cytokine driven) MΦ-0 with *E. coli* and analysed their anti-pathogenic capacity: Phagocytic indices (PI; Fig. 2A) and ROS production of PBMΦ-0 and CBMΦ-0 were similar (224.4 ± 44.3 in PBMΦ-0 vs. 221.7 ± 70.4 in CBMΦ-0). We as well detected no differences with regard to intracellular killing of bacteria (Fig. 2B). Starting from already higher HIF-1α concentrations in the non-infected state (compare Fig. 1D first and 4^th^ column), infected CBMΦ-0 up-regulated HIF-1α twice as much as PBMΦ-0 (Fig. 2C). Despite this finding, infected PBMΦ-0 up-regulated TLR4 to significantly higher levels than CBMΦ-0, with a high phosphorylation of NFkB P65 (Fig. 2D,E). The latter is a transcription factor; its phosphorylation leads to a cascade of pro-inflammatory reactions in bacterially infected MΦ-0^18^. Likewise, the secretion of IL6, tumor-necrosis-factor-alpha (TNFα), and IFNy was found higher in infected PBMΦ-0 (all p < 0.05 vs. CBMΦ-0, Fig. 2E–G).

**Figure 3:**
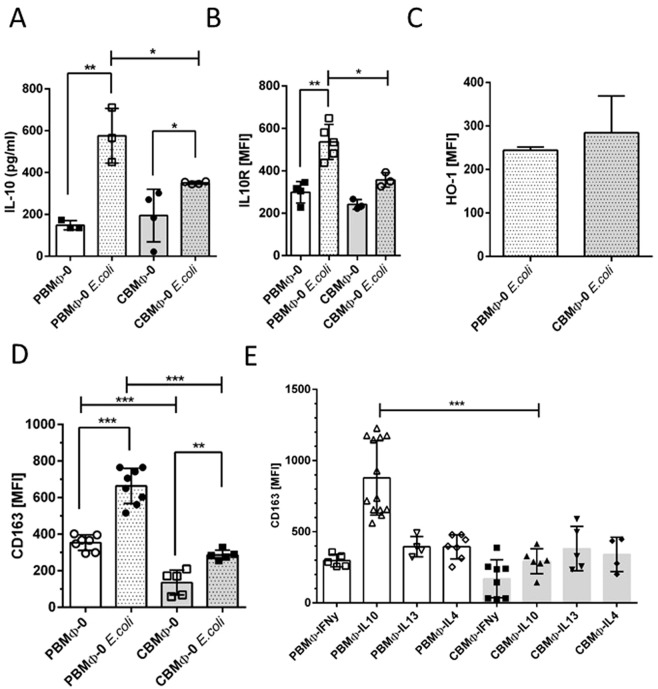
IL10 signaling and CD163 expression on MΦ-0 and MΦ-IL10. IL10 secretion was determined via ELISA (**A**). IL10R expression was assessed by FACS (**B**) Expression of HO-1 was measured 12 h p.i. (**B**). CD163 expression was quantified by FACS analysis of the indicated MΦ-0 groups (**D**,**E**,*p < 0.05, **p < 0.01, ***p < 0.005; forked bars, student’s t-test, blunt bars, two-way-ANOVA).

In non-infected PBMΦ-0 and CBMΦ-0, the secretion of IL10 was similar (Fig. 3A, first and third columns). After infection PBMΦ-0 and CBMΦ-0 both showed an increase in IL10 secretion, however, the increase was almost twice as high in PBMΦ-0 (Fig. 3A). For MΦ-IL10 polarisation, IL10 signaling is crucial^19^. The analysis of IL10-receptor (IL10R) expression after infection revealed that PBMΦ-0 up-regulated IL10R to a significantly higher level than CBMΦ-0 (Fig. 3B). Infection with *E. coli* did not enhance expression of HO-1, compared to the non-infected state (Fig. 1C).

IL10 is known to be a strong inducer of the scavenger receptor CD163^20,21^. Paralleled by the increase of IL10R and the IL10 secretion in infected PBMΦ-0 (Fig. 3A,B), we found a higher expression of CD163 in infected PBMΦ-0 (Fig. 3D), at a level comparable to non-infected PBMΦ-IL10. Polarisation with IL4 and IL13 did not lead to up-regulation of CD163 (Fig. 3E).

AKT phosphorylation occurred in PBMΦ-0 over twice as much as in CBMΦ-0 (Supplementary Fig. 3A), pointing to an engagement of mTOR. This activation pattern did not change in MΦ-IL10 (Supplementary Fig. 3B). We had shown before^11^ that mTOR signaling is reduced in CBMΦ. Rapamycin, an inhibitor of mTOR, did not reduce IL10 secretion of infected PBMΦ-0 but resulted in significantly lower expression of CD163 (Supplementary Fig. 2A,B). Taken together, these findings provide evidence that *E. coli* infection initiates comparable bactericidal reactions in PBMΦ-0 and CBMΦ-0 but differs in the PAMP dependent signaling, resulting in enhanced cytokine production.

### CBMΦ-IL10 show impaired clearing of senescent erythrocyte remnants

**Figure 4:**
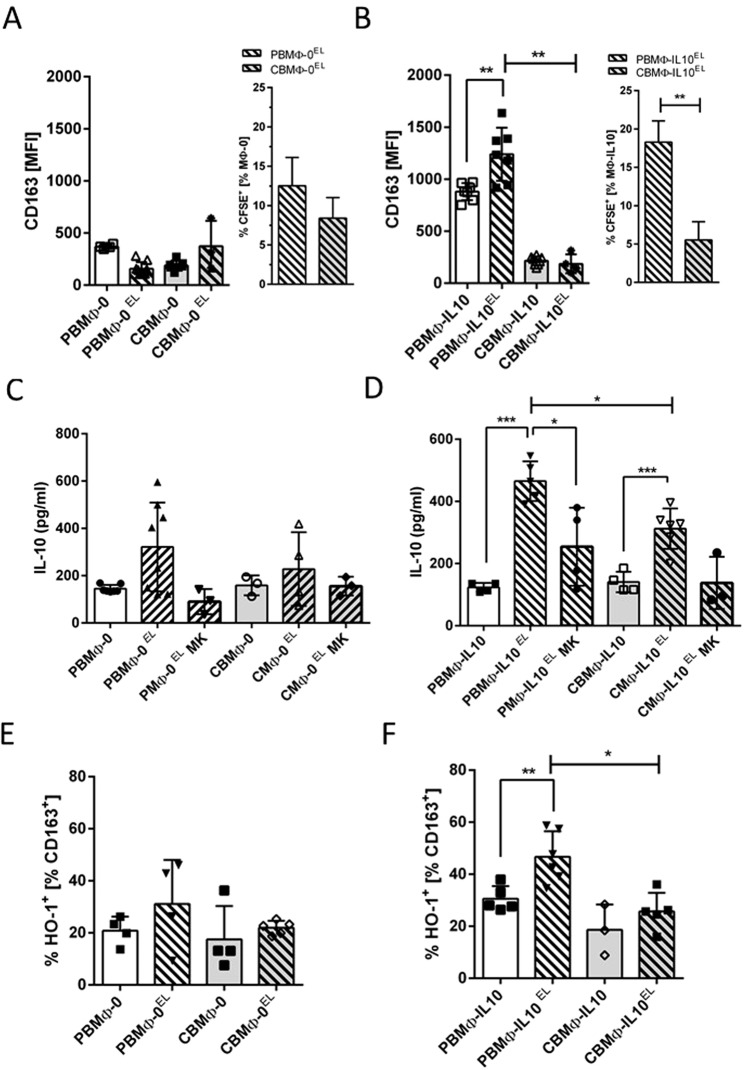
Regulation of CD163 expression, uptake of senescent erythrocytes (EL), IL-10 secretion, and assessment of HO-1 expressing MΦ. CD163 expression (**A**,**B**, left charts) and incorporation index (% of CFSE-positive MΦ) of senescent erythrocyte lysates (EL) were assessed 2 h post treatment. (**A,B**, right charts). IL-10 secretion from MΦ-0 (**C**) and MΦ-IL10 (**D**) was measured 24 h post treatment (*p < 0.05, **p < 0.01, ***p < 0.005, forked bars, student’s t-test, blunt bars, two-way-ANOVA).

We tested, whether the observed difference of CD163 expression on MΦ-0 and MΦ-IL10 (Fig. 3) would have functional consequences (Fig. 4). CD163 is involved in the clearance of Hb:Hp complexes, serum levels of which are elevated after birth, infection, trauma, and organ damage^22,23^. Therefore, we tested the capacity of MΦs to take up senescent erythrocytes (EL). We co-cultured MΦ-0 (Fig. 4A,C,E) and MΦ-IL10 (Fig. 4B,D,F) with carboxyfluoresceinsuccinimidylester- (CSFE-) marked senescent erythrocytes as a source for Hb:Hp complexes and analysed the uptake.

Expression of CD163 on PBMΦ-0 and CBMΦ-0 was not altered after co-cultivation with EL (Fig. 4A left). In contrast, CD163 was up-regulated only in the PBMΦ-IL10 subtype (Fig. 4B left). Whereas the erythrocyte uptake of MΦ-0 was not different (Fig. 4A right), CBMΦ-IL10 incorporated fewer erythrocytes than PBMΦ-IL10 (Fig. 4B right). CD163 plasma-membrane expression was elevated on PBMΦ-IL10 co-cultured with EL compared to PBMΦ-0. This process could not be observed in CBMΦ. These data suggest a functional correlation between lower CD163 expression on CBMΦ, lower IL10 secretion, and their reduced clearance of erythrocytes.

### CD163 expression correlates to IL10-secretion and HO-1 expression in PBMΦ-IL10 after phagocytosis of erythrocytes

Engagement of CD163 initiates PI3k/AKT signaling, which, again, leads to IL10 secretion by the cell^24–26^. PBMΦ-0 and CBMΦ-0 showed no significant differences in IL-10 secretion after co-cultivation with EL (Fig. 4C). For MΦ-IL10 a significant increase of IL-10 production was assessed 24 h p.i., but the concentration was significantly higher in PBMΦ-IL10 compared to CBMΦ-IL10 (Fig. 4D). Addition of the AKT inhibitor MK-2066 (MK) to EL treatment reduced IL-10 secretion in PBMΦ-IL10, suggesting that AKT signaling is engaged in this process. This finding was supported by phosphorylation of AKT in PBMΦ-IL10 and CBMΦ-IL10 (Supplementary Fig. 3D).

MΦ expressing HO-1 were not elevated in EL treated MΦ-0 (Fig. 4E). In contrast, more PBMΦ-IL10 expressed HO-1 compared to CBMΦ-IL10 (Fig. 4F). Taken together, the results suggest that an enhanced CD163 expression in PBMΦ-IL10 after EL exposure is attributed to increased IL-10 secretion and in turn HO-1 expression is up-regulated.

### T-cell stimulation and induction of FoxP3-positive regulatory T-cells under polarisation

**Figure 5:**
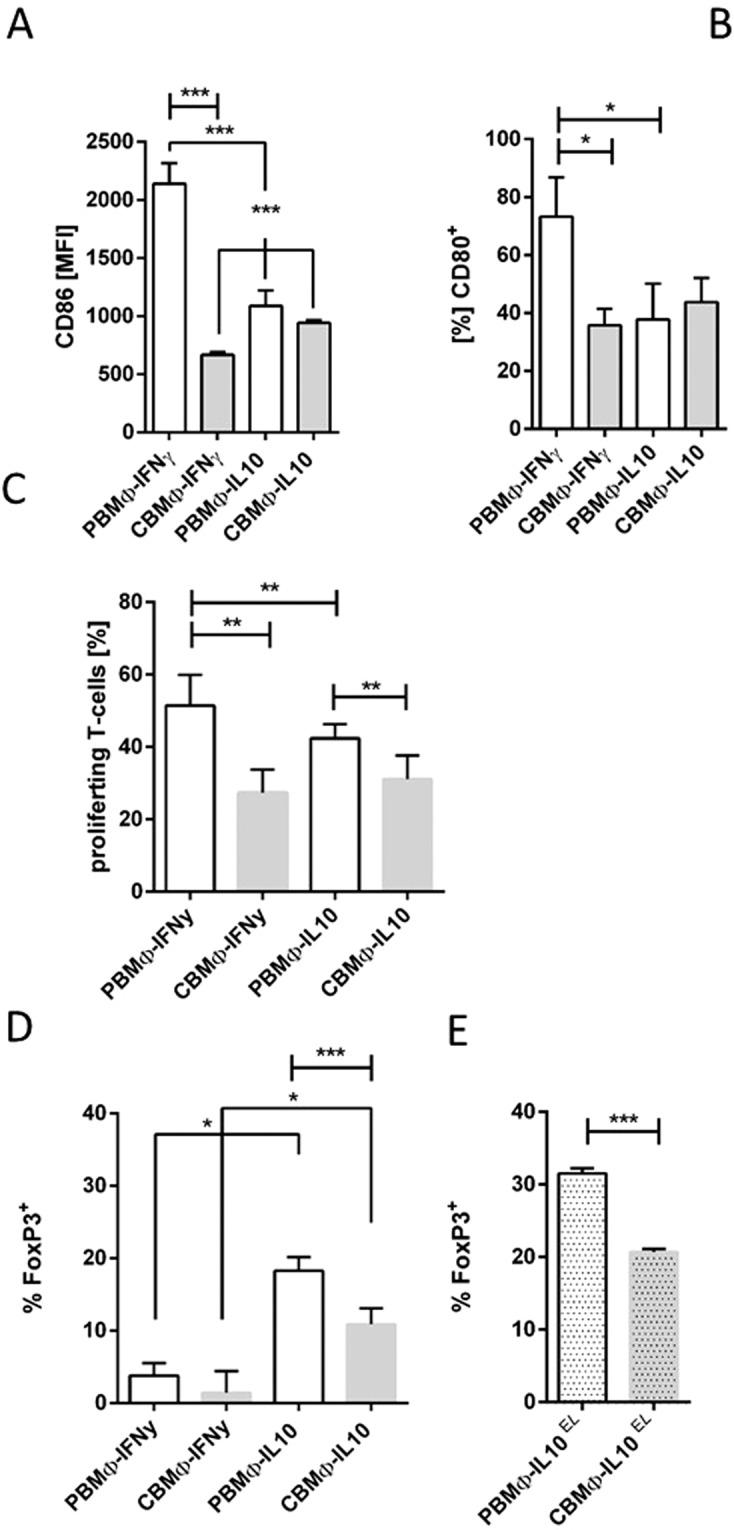
Polarisation of CBMΦ leads to alterations of B7 molecule expression, reduced T-cell blast formation and decrease of FoxP3 expressing T-cells. Expression of the B7-family molecules were assessed by flow cytometry for the indicated MΦ populations (**A**,**C**). Proliferation of non-infected T cells was measured by determination of T-cell blasts (**B**; decrease of mean CFSE fluorescence intensity as depicted in Supplementary Fig. 1D). Regulatory T-cells were characterized by intracellular FoxP3 staining within the population of CD4 positive lymphocytes (**D**). FoxP3 expressing T-cells were also quantified after addition of senescent erythrocytes (**E**; all charts n = 3; *p < 0.05, **p < 0.01, ***p < 0.005, student’s t-test, simple bar, forked bars, two-way-ANOVA).

T-cell proliferation requires a variety of co-stimulatory molecules, e.g. CD80 and CD86, members of the B7-family. We assayed the surface expression of CD86 on polarised MΦ and found a significant reduction on CBMΦ-IFNy and CBMΦ-IL10 (Fig. 5A) compared to corresponding PBMΦ. In line with this finding we detected more CD80 expressing PBMΦ-IFNy (Fig. 5B). Since these receptors influence antigen presenting cell-dependent T-cell stimulation, we co-cultured polarised MΦ with T-cells and stimulated with αCD3 mAb for 72 hours, as shown before^27^. We found adult T-cell blast formation profoundly reduced, when CBMΦ-IFNy and CBMΦ-IL10 were co-incubated as a source of co-stimulatory signals. Cord blood T-cell proliferation was diminished, as already known. (Fig. 5C).

Among other signals, IL10 is crucial for the expansion of regulatory T cells (Tregs^28^). We analysed the induction of FoxP3 among CD4^+^ T cells (Fig. 5D). Whereas MΦ-IFNy subtypes induced equivalent proportions of FoxP3 expressing T cells, their induction under IL10 polarisation was significantly augmented by PBMΦ (p < 0.05 vs. CBMΦ). EL treatment enhanced the expansion of FoxP3^+^ Tregs in both PBMΦ-IL10 and CBMΦ-IL10 (Fig. 5E).Taken together, the reduced expression of B7 molecules on polarised CBMΦ compared to PBMΦ may be attributed to lower T cell expansion and a reduced Treg population in CBMΦ.

## Discussion

Like circulating monocytes, MΦ play a central role in shaping the immune response by production of bactericidal molecules, cytokines and by their phagocytic activity. Since neonatal sepsis is often followed by inflammatory organ damage, and since local tissue MΦ are extremely difficult to obtain, we decided to polarise monocyte-derived macrophages by preconditioning with M-CSF and additional treatment with either IFNy, or IL10. These microenvironments mirror the acute inflammatory phase as well as the resolving phase of infection and may help to decipher the functional differences of neonatal MΦ *in-vivo*.

In our previous publication, CBMΦ (MΦ-IFNy, MΦ-IL10) were characterized by a reduced capability to express MΦ-IL10 driven receptor signaling cascades^11^. Our present data suggest that neonatal CBMΦ-0 polarise aberrantly due to a crucial role for deficient intracellular signal transduction via STAT1, STAT3, NFkB, and HO-1 (Figs. 1, 2): In PBMΦ-0, the pSTAT3/pSTAT1 balance outweighs pSTAT1 and concomitantly shifts polarisation towards a non-inflammatory status. This balance is compromised in CBMΦ-0, which may be attributed to a lower susceptibility to the M-CSF signal, due to a tolerance generated by elevated M-CSF levels during pregnancy^29,30,^ although M-CSF concentrations of the current *in-vitro* polarisation protocol were chosen to be high enough to level out these differences.

HO-1, which classifies as an anti-inflammatory factor^31^, was equally expressed in PBMΦ-0 and CBMΦ-0 (Fig. 1D). HIF-1α is regarded as a pro-inflammatory key molecule^32^; its significantly stronger expression in CBMΦ-0 extenuated the anti-inflammatory bias of HO-1 expression (Fig. 1C). With the exception of IL6, the basal cytokine expression was similar in PBMΦ-0 and CBMΦ-0 (Fig. 2E–G).

IFNy skewed the pSTAT3/pSTAT1 balance in PBMΦ-IFNy but failed to do so in CBMΦ-IFNy (Fig. 1A,B, second vs. third columns), confirming earlier results^33^. CBMΦ-IFNy exhibited high HIF-1α expression but an inadequate pSTAT1 activation (Fig. 1A,C). This can be caused by a crosstalk via IL6 and pSTAT3^34^. The aberrant pSTAT3 up-regulation in CBMΦ-IL10 resulted in a diminished HO-1 expression (Fig. 1D), concordant with previous results supporting the hypothesis for a diminished anti-inflammatory polarisation of CBMΦ^11^.

Addressing the question whether naïve CBMΦ-0 reacted on pathogen-associated-molecule-pattern (PAMP) comparable to PBMΦ-0, we found no significant differences in bacterial killing (Fig. 2A,B), but a diminished secretion of both, pro- (i.e. TNFα and IL6; Fig. 2E–G) and IL10 (Fig. 3B). Studies with LPS activated PBMΦ^35^ show similar results as we have seen with *E. coli* infected PBMΦ-0. The reduced cytokine production in CBMΦ-0 is caused by a weaker NFκB and TLR4 response (Fig. 2D,E). A diminished IL10 sensitivity towards IL10 in CBMO^12^, and a reduced production in CBMΦ after LPS treatment, support our observation^36^. To our knowledge, we showed for the first time that the IL10R is down-regulated on the plasma-membrane of CBMΦ-IL10, suggesting that IL10-/IL10R-signaling is dampened (Fig. 3B,C).

Compared to the non-infected MΦ-0, *E. coli* challenged CBMΦ-0 upregulated HIF-1α more strongly compared to PBMΦ-0 (Fig. 2C, compare to Fig. 1C, first and 4^th^ columns). HO-1 was not up-regulated after *E. coli* infection (Fig. 3C, compare to Fig. 1D, first and 4^th^ columns), although HIF-1α up-regulates HO-1 expression to enable the transition from pro- to anti-inflammatory reactions^37^. To deploy its function, HIF-1α requires IL10^38^, which we found reduced in *E. coli* challenged CBMΦ-0.

*E. coli* infection is attributed to IL10 secretion and CD163 up-regulation in PBMΦ-0 and PBMΦ-IL10 (Fig. 3A,D). CD163 was not up-regulated in MΦ-IL4 and MΦ-IL13 (Fig. 3E). In contrast, addition of IL-10 to CBMΦ had no effect on CD163 expression, most likely due to a scarcity of the IL10R (see above, Fig. 3D).

The sequestration of iron and derivatives is a crucial function of MΦ, depending from the transient polarisation status, since iron is an energy source for pathogens^38^. Iron containing hemoglobin is a product of inflammation, causes hemolysis and is bound by haptoglobin^39^. The Hb:Hp complexes in turn are bound and internalized by CD163^40^. We, therefore, compared the capacity of MΦ-0 as well as MΦ-IL10 (Fig. 4).

The detection of comparable erythrocyte uptake rates in PBMΦ-0 and CBMΦ-0 (Fig. 4A) can be explained by CD163 independent phagocytosis mediated by Fc-y receptors. The expression of CD163 and functional erythrocyte uptake could be seen for PBMΦ-IL10 and CBMΦ-IL10 (Fig. 4B). Comparing the IL-10 secretion and HO-1 upregulation of MΦ-0 and MΦ-IL10 upon EL challenge (Fig. 4C,F), we found more PBMΦ-IL10 than CBMΦ-IL10 to secrete IL10. Moreover, a higher percentage of PBMΦ-IL10 upregulated HO-1 after EL challenge, suggesting a CD163 driven signaling to anti-inflammatory polarisation in adults, which is diminished in newborns. CD163 dependent HO-1 regulation could be a target for therapeutic strategies, since HO-1 reduces oxidative stress, which is one of the main triggers for bronchopulmonary disease (BPD)^41^.

AKT dependency for IL10 secretion was also demonstrated by its pharmacological inhibition, utilizing MK2260. IL10 secretion was inhibited, but not entirely blocked (Fig. 4D, 4^th^ and 6^th^ column). The fact, that AKT signaling is not entirely linked to CD163 expression can be explained by the antagonistic function of the AKT isoforms AKT1 and AKT2 respectively^42^.

PBMΦ-IFNy but not CBMΦ-IFNy responded with increased presentation of B7-family molecules, resulting in an enhanced proliferation of T-cells (Fig. 5A–C). The reduced capacity of CBMΦ-IFNy to activate T-cells already has been demonstrated for CBMO^27^.

Regulatory T-cells (Tregs) control the immune response by supressing pro-inflammatory reactions. We determined a higher percentage of FoxP3-positive T-cells in PBMΦ-IL10 polarised cultures (Fig. 5D). Our observation differs from findings in non-polarised conditions: In cord blood, suppressive Tregs were found increased compared to adult blood in the early phase after birth^43^ and may expand under inflammatory conditions, e.g. necrotizing enterocolitis (NEC)^44^.

We interpret the results of the manuscript carefully: Since they are based on an *in-vitro* polarisation protocol, the concentrations of cytokines, the density of *E. coli* and erythrocyte lysates for the analysis of MΦ functions may only partially highlight differences. Since CD71^+^ erythroid cells and MDSCs were reported to antagonize pro-inflammatory and bactericidal reactions, they could also contribute to the aberrant CBMΦ polarization, described here^13,45^. The *in-vitro* infection with *E. coli* neither exhibited a reduced phagocytosis nor resulted in an excessive survival of bacteria (Fig. 2A,B), suggesting that MDSC and erythroid cells did not downregulate this function and may not play a dominant role regarding to periphagocytic reactions. Future experiments are planned to elucidate this question.

Although a fast and effective systemic inflammatory response is a prerequisite against microbial invasion, it should resolve as soon as the microbes are eliminated. Delay or failure of inflammation-resolution processes leads to dysregulated and prolonged inflammation, which can damage organs and contribute to the development of malignant diseases, chronic lung disease, rheumatoid arthritis, type-2 diabetes mellitus, heart disease, and neurological disorders in adults^46^. It is known that sepsis-induced sustained inflammation may initiate and perpetuate brain damage of preterm infants via cytokine production and neuronal apoptosis^10^. The inflammatory response includes reactions that normally already contain components for its termination. This resolution is tightly regulated by effector cell apoptosis^11,47^ and anti-inflammatory proteins, e.g. IL10 and transforming growth factor-β (TGF-β). Not only do preterm infants appear to have a paucity of some of these anti-inflammatory factors^48^, but moreover their functional and metabolic response to IL10 is diminished^11^, leading to functional consequences^12^ and a persistence of inflammatory cytokine production. Sustained neonatal systemic inflammatory response is associated with poor postnatal growth among infants born very preterm during the first year of life^49^.

The observations seen here, with a deficiency of cord blood monocytes to polarise into anti-inflammatory macrophage subtypes, together with a reduced induction of inhibitory Tregs, would blend into this model of sustained inflammation in neonatal sepsis and may offer new therapeutic targets.

## Materials and Methods

### Patients

The study protocol was approved by the Ethics Committees of Aachen University Hospital (Permission No: EK150/09, Oct. 6, 2009, signed by Profs G. Schmalzing and U. Buell). All adult participants involved were informed and gave written consent to use their blood samples for this study participation. All term neonates were delivered spontaneously and did not exhibit signs of infection, as defined by clinical status, white blood cell count and C-reactive protein. Mothers with amnion infection and prolonged labour (>12 hours) were excluded. Umbilical cord blood was placed in heparin-coated tubes (4 IE/ml blood), immediately following cord ligation as described before^50^. All methods were performed in accordance with the relevant guidelines and regulations.

### Reagents

Antibodies to CD4 (RPA-T4), CD80 (clone L307.4), CD86 (clone FUN 1), CD163 (clone GH1/61), HLA-DR (clone G46-2.6), TLR4 (clone HTA125), IL10 (clone JES3-9D7), FoxP3 (PCH101), TNFα (clone Mab11), HO-1 (clone MA1112), HIF-1α (polyclonal goat IgG), CD71 (LO1.1) and Ig-matched controls (IgG1, IgG2b) were from BD Biosciences (Heidelberg, Germany), eBiosciences (Frankfurt, Germany) and Immunotools (Friesoythe, Germany). Isopropyl-β-D-thiogalactopyranoside (IPTG) and antibiotics were purchased from Sigma (Munich, Germany). Staining was performed according to the manufacturer’s recommendations. Cytokines IFNy, IL4, IL10, M-CSF were from PAN Biotech (Aidenbach, Germany). CFSE was purchased from Molecular Probes (Eugene, OR, USA). RPMI was from Gibco (Paisley, UK). Green-fluorescent-protein (GFP) expressing *E. coli* were grown and used in infection assays as described before^50^. The phagocytosis index (CD14+ GFP+ MΦ %: CD14+ MΦ %) and the phagocytic capacity (mean fluorescent intensity (MFI) of CD14+ monocytes) were assessed by flow cytometry after 4 h p.i. (post infection) and 24 h p.i. For bacterial phagocytosis assays a multiplicity of infection of 25 (MOI25) was utilized. The AKT inhibitor MK 2206 was provided by Sellekchem and added 60 min before stimulation of cells at a final concentration of 1 µM.

In our experiments, we could not observe significant differences in bacterial killing, which could be due to the fact that we used polarised macrophages and the studies cited before conducted experiments with monocytes. As a control for purification, we stained for CD71^+^: The MΦ populations generated *in-vitro* only contained few CD71^+^ positive cells as revealed in FACS based staining. Unpurified mononuclear cells from cord blood contained about 50% erythroid cells (57.35% ± 24.34), whereas CBMΦ-IL10 only contained 8% erythroid cells (8.22% ± 1.9) on day 5.

### Differentiation protocols

Our polarisation protocol followed the protocols published earlier^1^. In brief, leukocytes were prepared by density gradient centrifugation of whole blood from healthy adult donors and cord blood from term neonates as described before^50^. Polarisation was induced by seeding 5 × 10^5^ cells/ml in 12-well tissue culture cells and administration of 100 ng/ml MCSF for 72 h in RPMI (designated as MФ-0). Where indicated further polarisation was achieved by addition of 50 ng/ml IFNy (designated as MФ-IFNy), 10 ng/ml IL4 (designated as MФ-IL4) or 10 ng hIL10 (designated as MΦ-IL10) for additional 48 h. For T-cell polarisation assays the cell number was adjusted to 5 × 10^6^ cells/ml in 6-well culture plates.

T-cell proliferation assays were performed as published^27^. In brief, lymphocytes were isolated and split into two groups. One group was left untreated while the other group was subjected to the differentiation protocol as described^11^. Then, untreated, syngeneic lymphocytes were co-cultivated with the MΦ population as indicated in presence of stimulatory antibody OKT-3 for two additional days before analysis.

### Stimulation of MΦ with senescent erythrocyte lysates

After Ficoll density separation, layers containing leucocytes, erythrocytes and serum were separated. The leukocyte suspension was subjected to the macrophage polarisation procedure described above. The erythrocyte fraction (80% hematocrit) was submitted to a second centrifugation step of 10,000 × g for 15 min. The lower 10% fraction was collected representing senescent erythrocytes^51^, which were resuspended in the autologous serum and further diluted 1:1 in phosphate bufferd saline (PBS).

The senescent erythrocyte/serum suspension was stored at 4 C° for 5 days. Afterwards the erythrocyte/serum suspension was centrifuged (300 × g, 15 min). The supernatant was stored for later usage. Pelleted erythrocytes were stained with CFSE according to standard protocols. After washing to remove excessive CFSE, labelled erythrocytes were resuspended in the supernatant collected before the CFSE staining procedure.

Polarised macrophages were detached from culture plates by adding PBS/EDTA (10 mM EDTA v/v, 10 min). The cell count of the CFSE-stained erythrocytes and the macrophage was measured using flow cytometry (FACS Canto II, see below), thereby controlling CFSE staining efficacy. MФ and CFSE-stained erythrocytes were mixed in a ratio 1:100 and further cultivated under standard conditions (RPMI, 10% v/v FCS, 37 C°, 5% CO2). It was previously reported that senescent erythrocyte suspensions release Hb and Hb:Hp compexes^52^. Phagocytosis and peri-phagocytic reactions were assessed as described in the result section. Co-cultivation of MΦ with senescent erythrocyte lysates were designated with the abbreviation EL.

Intracellular intermediates, cytokine and transcription factor detection.

For detection of reactive-oxygen-species (ROS), the ROS detection kit (eBiosciences, Frankfurt, Germany) was used according to the manufacturer’s recommendations. Cells were fixed in 1% paraformaldehyde for 1 hour and permeabilized with 0.1% v/v Triton-X100 in PBS for 5 min. Afterwards cells were washed and blocked with PBS/FCS (5% v/v) for 20 min at room temperature (RT). Cells were washed again and stained with fluorochrome labelled antibodies in PBS/FCS (5% v/v) for 60 min at RT followed by additional washing. Intracellular staining of human peripheral blood mononuclear cells (PBMCs) with PCH101 antibody was done using the anti-human Foxp3 staining set from eBioscience following the manufacturer’s recommendations.

### Flow cytometry

A daily calibrated FACS-Canto flow cytometer (Becton Dickinson, MountainView, CA) was used to perform phenotypic analysis. To prevent nonspecific binding, cells were incubated with 10% fetal calf serum on ice for 10 minutes before staining with pacific-blue (PB)-, fluorescein-isothiocyanate (FITC)-, phycoerythrin (PE)-, allophycocyanin (APC)-, or anti-IgG secondary -labelled monoclonal antibodies for 20 minutes on ice in the dark. Monocytes were gated using forward scatter (FSC), side scatter (SSC), and CD14 expression. The gating strategy and typical analysis is provided in Supplemental Fig. 1.

### ELISA

The TNFα and IL10 enzyme-linked immunosorbent assays (ELISA) were purchased from eBbiosciences (Ebiosciences-Natutec, Frankfurt, Germany) and used according to the manufacturer’s recommendations. The IL6 ELISA was purchased from Immunotools (Friesoythe, Germany). The read-out was executed in a spectra max 340PC ELISA reader (molecular devices, Sunnyvale, CA, USA) with a sensitivity from 4–500 pg/ml.

### Western blot

For the immunoblot analysis, 6 × 10^6^ cells were subjected to SDS-PAGE which was performed according to standard protocols. For imaging and quantification, a LAS 3000 imager (Fujifilm, Düsseldorf, Germany) combined with the Multi-Gauge software (Fujifilm, Düsseldorf, Germany) was used.

### Statistical analysis

Results are expressed as mean +/− standard deviation. Error bars represent standard deviations. Values of p < 0.05 were considered significant. Analyses were done with statistical software performing student’s t-test and two-way ANOVA. Experiments with N = 3 were tested according to Mann-Whitney for significant difference. Data which did not pass a test for Gaussian distribution were tested with a Kolmogorov-Smirnov test as provided by Graph Prism Pad Software Statistical Package, La Jolla, CA 92037 USA.

## Supplementary information

Supplementary Information .

## Data Availability

The datasets generated during and/or analysed during the current study are not publicly available but are available from the corresponding author on request. The authors declare to make materials, data and associated protocols promptly available to readers after acceptance of the manuscript and will upload all original data to a repository.
