# Supplementary material for: Impaired functional capacity of polarised neonatal macrophages
Source: Sci Rep. 2020 Jan 17;10:624. doi: 10.1038/s41598-019-56928-4 (PMC6968972; doi:10.1038/s41598-019-56928-4)
Supplement: Supplementary file 1 — Supplementary Information . [file 41598_2019_56928_MOESM1_ESM.pdf]

Supplementary Files to: SREP19 36703A

Impaired functional capacity of polarised neonatal macrophages

Running title: monocyte polarisation in cord blood

Authors: Stephan Dreschers[1], Kim Ohl[2], Nora Honke[2], Klaus Tenbrock[2], Thorsten W. Orlikowsky[1]

Supplementary Figure 1

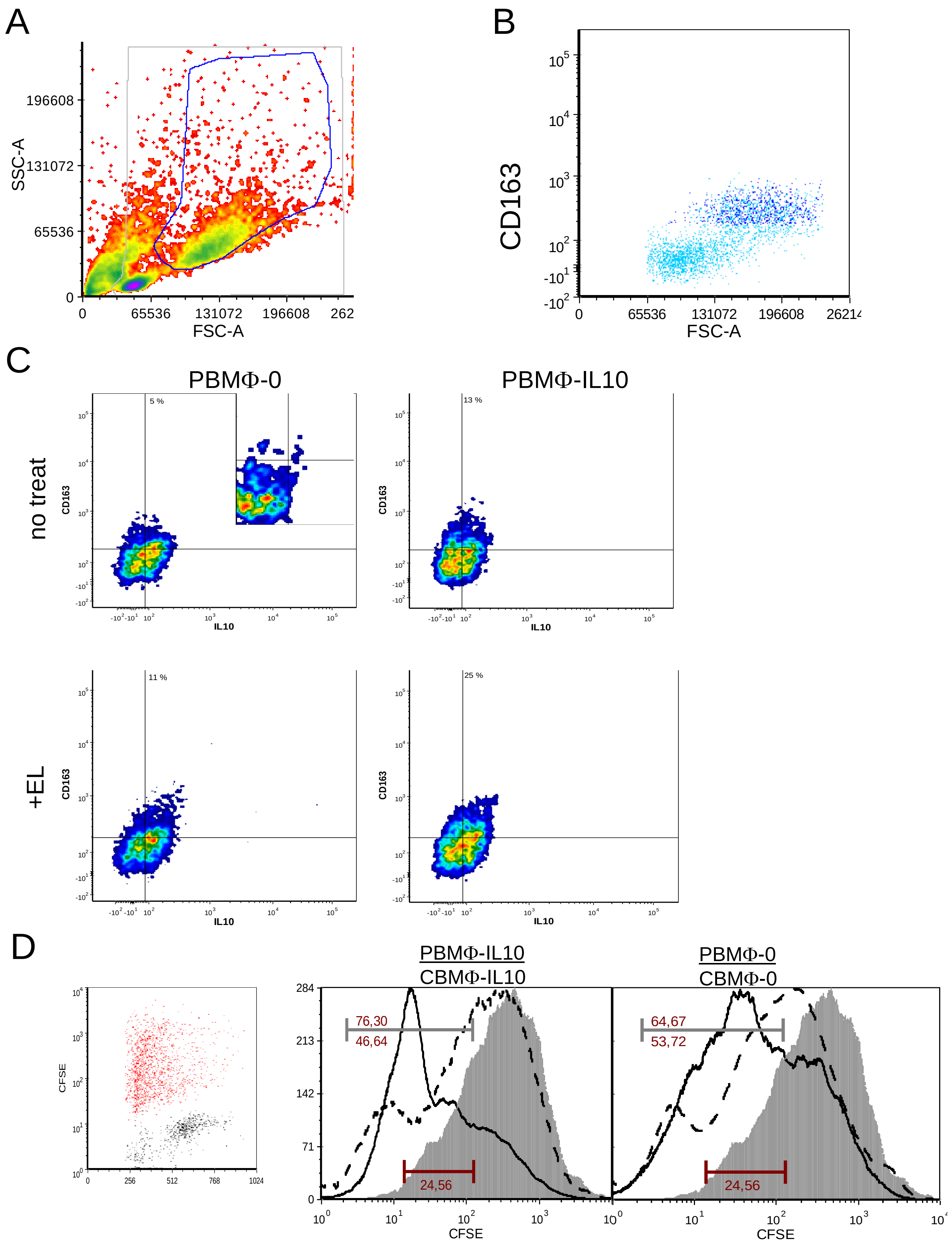

Supplementary Figure 1: Gating strategy and dot plot analysis. A typical FCS-A/SSC-A density plot of MΦ-0 (A). Grey lines show the intact lymphocyte gate, blue lines the monocyte/macrophage gate. CD163 staining (B) comparing the isotype control (light blue) and the CD163-positive MΦ fraction (dark blue). Typical example of CD163/IL10 double-staining of indicated PBMΦ (C) before and after stimulation as summarized in Fig.4 D -H. The small integrated density plot depicts the double isotype control. OKT-3 induced MΦ proliferation is depicted in (D). The dot plot shows the CFSE labelling control of MΦ. The histograms show the CFSE intensity representing different generations of proliferating MΦ with indicated polarisation (dotted lines, CBMΦ; bold lines, PBMΦ; filled profile, CFSE labelled and OKT-3 stimulated PBMΦ on day 1 as control).

Supplementary Figure 2

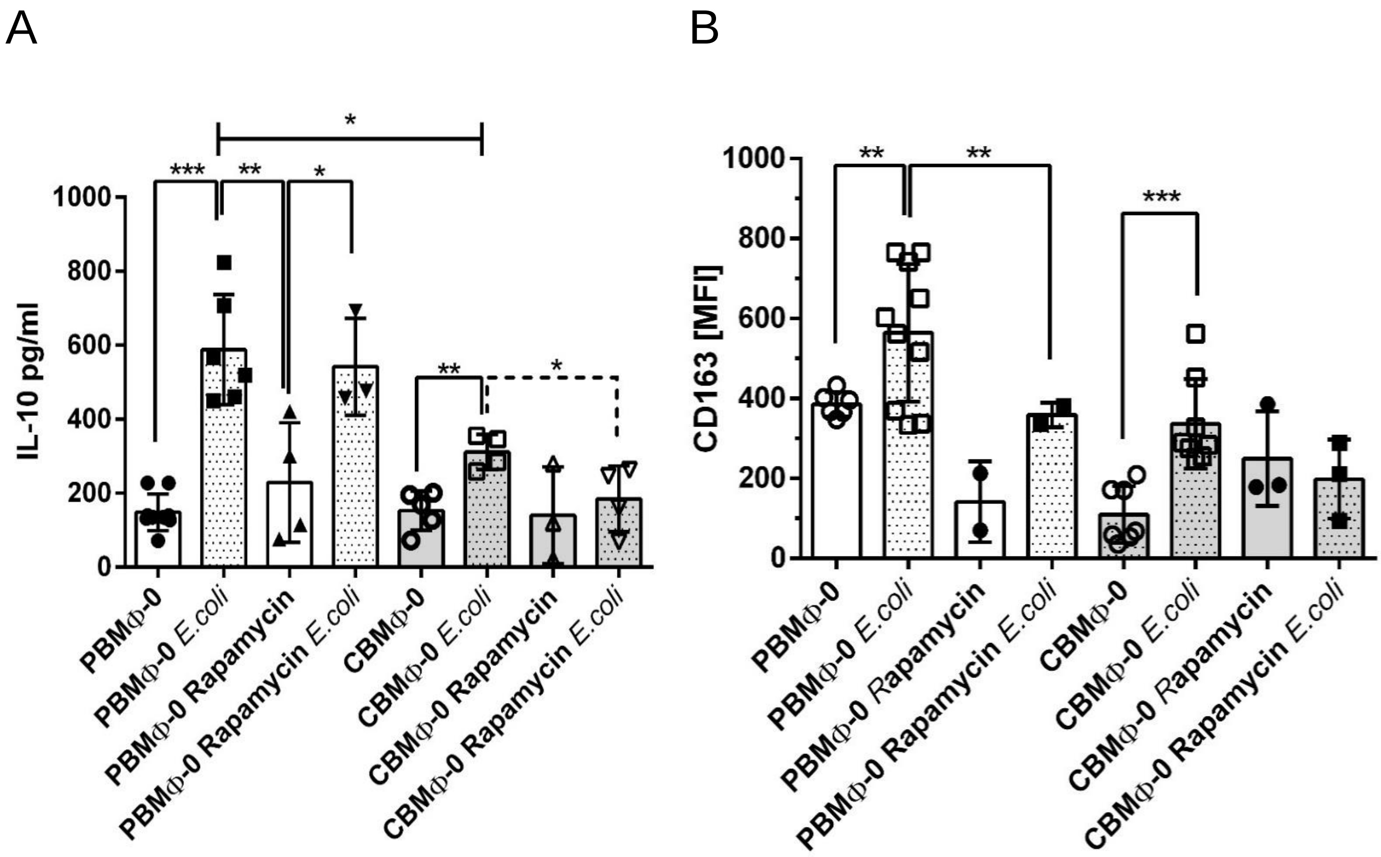

Supplementary Figure 2: IL10 secretion and CD163 expression upon treatment with rapamycin. ELISA results of MΦ are given in (A). CD163 receptor densities were assessed by flow cytometry and summarized in (B; * $p < 0.05$, **, $p < 0.01$, ***, $p < 0.005$, forked bars, student`s t-test, simple bars one-way-ANOVA and blunt bars, two-way-ANOVA).

Supplementary Figure 3

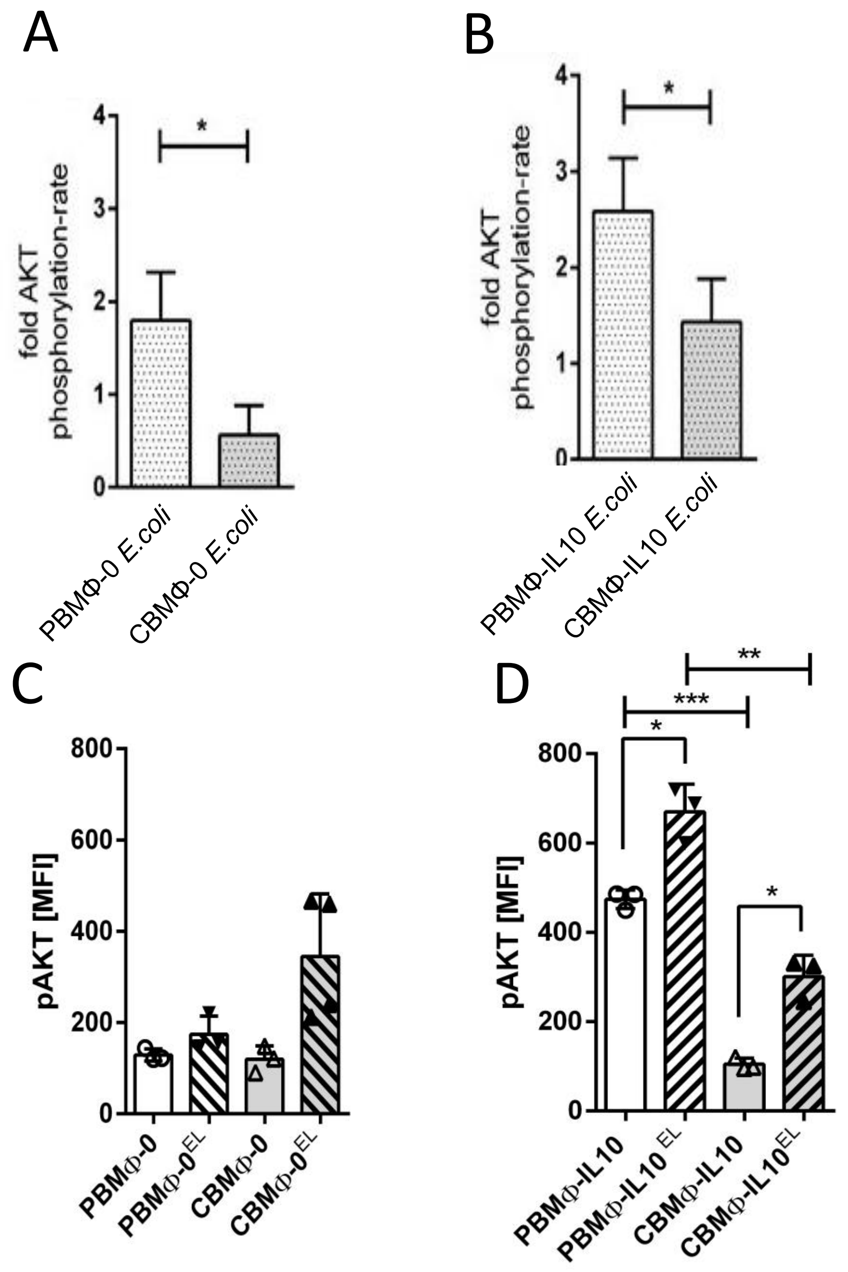

Supplementary Figure 3: AKT activation via phosphorylation in exposed MΦ is not linked to CD163 internalization. Phosphorylated AKT was detected on immunoblots after *E.coli* (A, B) and EL (C, D) co-cultivation in indicated subsets (* $p < 0.05$, **, $p < 0.01$, ***, $p < 0.005$, forked bars, student`s t-test, simple bars one-way-ANOVA and blunt bars, two-way-ANOVA).
